# Tracheobronchopathia Osteochondroplastica—Clinical, Radiological, and Endoscopic Correlation: Case Series and Literature Review

**DOI:** 10.1177/2324709620921609

**Published:** 2020-05-14

**Authors:** Carlos Alejandro García, Saveria Sangiovanni, Valeria Zúñiga-Restrepo, Eliana Isabel Morales, Luz Fernanda Sua, Liliana Fernández-Trujillo

**Affiliations:** 1 Fundación Valle del Lili, Cali, Colombia; 2 Universidad Icesi, Cali, Colombia

**Keywords:** tracheobronchopathia osteochondroplastica, bronchoscopy, tracheal diseases, case report

## Abstract

Tracheobronchopathia osteochondroplastica (TO) is a rare idiopathic and benign disease that is often underdiagnosed. TO is characterized by multiple submucosal cartilaginous and osseous tracheobronchial nodules that spare the posterior wall. It usually affects the elderly, developing when the person is around 60 years old without gender preference and has a reported incidence of 0.11%. TO can be symptomatic and should be considered in patients with chronic cough, dyspnea, and recurrent pulmonary infections. Diagnosis is usually incidental by computed tomography or bronchoscopy, the latter being the gold standard diagnostic test for TO. Many thoracic imagers are not well acquainted with TO; thus, these patients are often underdiagnosed or misdiagnosed. We came across 5 patients in our institution who were incidentally diagnosed with TO, inspiring us to review the available literature on this disease. A total of 33 patients diagnosed with TO between 2009 and 2019 were identified by our retrospective review. Clinical and imaging data were collected on these patients. We also included the clinical, radiological, and endoscopic data of our 5 cases. TO should be considered in patients with chronic cough, dyspnea, and recurrent pulmonary infections. Our experience is that both computed tomography and bronchoscopy can be used to make a reliable diagnosis. It is crucial for physicians, especially radiologists and pulmonologists, to be aware of the existence of TO in order to ensure proper diagnosis.

## Background

Tracheobronchopathia osteochondroplastica (TO) was first described in 1855 by Rokitownski.^1^ It is an idiopathic, benign, rare, and underdiagnosed disease that causes stenosis, mainly in the trachea, sometimes with affection of the larynx and main bronchi.^2^ TO presents multiple submucosal cartilaginous and osseous tracheobronchial nodules in the anterior and lateral walls, over the cartilaginous rings, preserving the posterior membranous wall.^2-4^ Although its etiology has not been elucidated, TO might be associated with cartilaginous outgrowth with latter ossification, elastic tissue metaplasia, atrophic rhinitis, pulmonary infections, amyloidosis, lung cancer, among others.^1,5^ No relationship has been established between TO and calcium or phosphorus metabolism.^1^ Its incidence has been reported around 0.11%, usually presenting at 60 years old, with no gender preference. However, epidemiologic data are scarce, and since TO is usually asymptomatic or presents with nonspecific symptoms, a better characterization of the disease is needed.^3^ Patients with TO usually have an unremarkable chest physical examination as well as a normal chest radiograph; thus, its diagnosis is usually incidental. A diagnostic algorithm should include chest computed tomography (CT) scan and bronchoscopy.^4,6^ Many thoracic images are not well acquainted with TO; therefore, these patients are misdiagnosed. We believe that a better understanding of the disease and improvement in recognition of its signs will lead to an early and adequate diagnosis. In this article, we aim to present a clinical, imaging, and endoscopic correlation of 33 patients found in the literature and 5 patients from our hospital, emphasizing on diagnostic approach.

## Case Presentation

### Case 1

**Figure 1.**
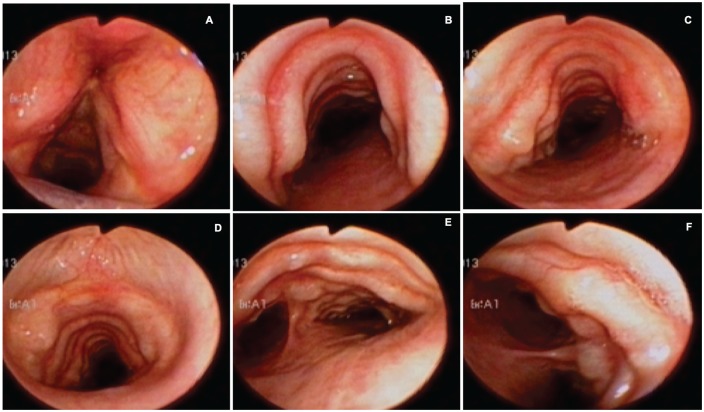
Bronchoscopy. (A) Vocal cords of normal aspect. (B and C) Superior trachea with irregular nodular lesions over cartilaginous rings, with greater profusion toward the left side, covered by intact mucosa. (D) Middle third of the trachea with similar lesions and mild decrease of tracheal lumen. (E) Inferior third of the trachea with greater profusion of lesions near the carina, sparing completely the posterior wall. (F) Right main bronchus with similar nodular lesions in the lateral wall.

A 79-year-old man, with a history of arterial hypertension, coronary artery disease, atrial fibrillation, and heart failure, admitted due to a clinical course of several days of fever, dry cough, dyspnea, and malaise. Chest radiograph showed multiple infiltrates in the right middle lobe and pneumonia was suspected, although no etiology was found at the moment. Bronchoscopy was performed and multiple nodules were found from the subglottic area to the main bronchi, distributed in the anterior and lateral walls with “cobblestone throat” appearance (Figure 1).

### Case 2

**Figure 2.**
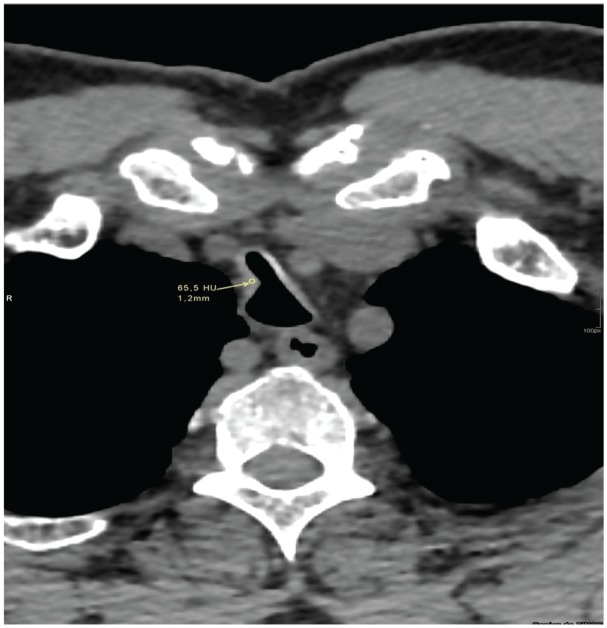
Thoracic axial computed tomography scan. Loss of normal tracheal morphology with soft tissue nodule (65 HU) on the right tracheal wall, without significant stenosis.

A 60-year-old woman, admitted due to laryngeal stridor and dyspnea after a history of 2 months of dry cough that had turned productive, accompanied by odynophagia, headache, malaise, and temperature of 37.5°C. She had a history of 10 years of active and passive smoking and renal transplantation 5 years prior to consultation on immunosuppressive and tuberculosis (TB) treatment with positive sputum smear microscopy. A thoracic CT was performed showing mixed interstitial alveolar infiltration on the superior and inferior areas of the right lower lobe. Multiple soft tissue nodules were found in the trachea, without stenosis (Figure 2). Bronchoscopy showed multiple whitish lesions in the lower third of the trachea and main bronchi, with a random distribution affecting the anterolateral walls. There were signs of left endobronchitis, and for this reason, endobronchial tuberculosis was ruled out.

### Case 3

**Figure 3.**
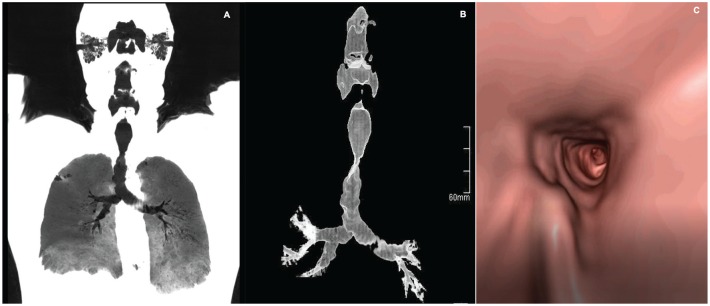
(A) Thoracic coronal computed tomography maximal intensity projection (CT MIP) reconstruction. Multiple endobronchial nodules are shown, along with reduced lung volumes. (B) Thoracic coronal CT MIP reconstruction. Irregular tracheal walls with moderate stenosis in the middle third. (C) Virtual bronchoscopy showing endotracheal nodular surface with rock garden appearance.

**Figure 4.**
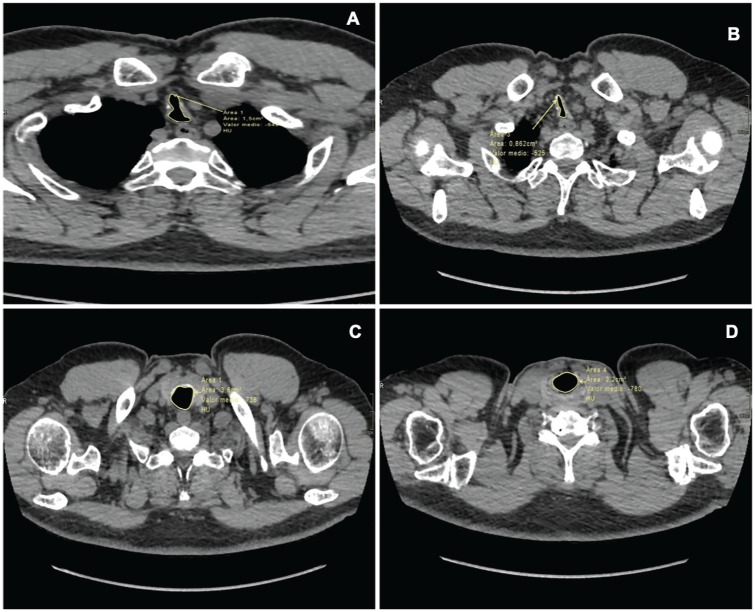
Thoracic axial dynamic computed tomography scan. (A) Demonstrating tracheal stenosis during inspiration with an area of 1.5 cm^2^ that reduces to (B) an area of 0.8 cm^2^ during expiration. (C) Compared with the superior third of the trachea with an area of 3.6 cm^2^ during inspiration and (D) 3.2 cm^2^ during expiration, which gives a stenosis of 75%.

**Figure 5.**
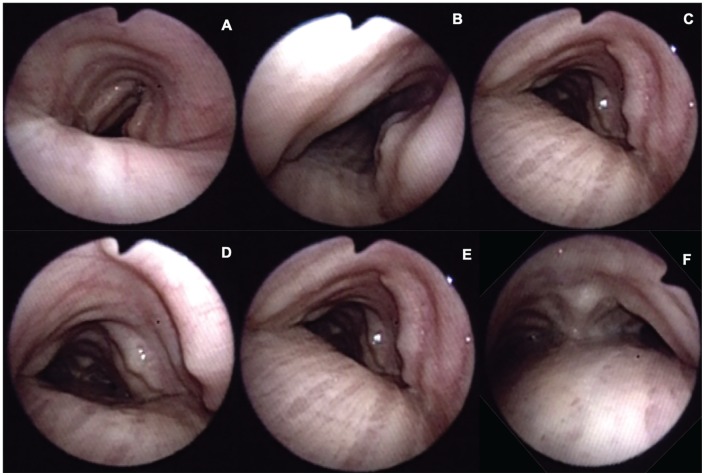
Bronchoscopy. (A and B) Middle third of the trachea where the deformity of the structure can be seen secondary to the nodules found on the anterolateral walls. (C-E) Inferior third of the trachea with random distribution of nodules that do not cause tracheal obstruction. (F) Main bronchi compromised by similar lesions.

A 63-year-old man, with a history of smoking, chronic dry cough, and recurrent pulmonary infections over the past 10 years. Chest CT was performed finding multiple endotracheal nodules on the right lung, along with reduced lung volume (Figure 3A). On maximal intensity projection reconstruction, irregular tracheal walls with moderate stenosis in the middle third were found (Figure 3B). Virtual bronchoscopy showed a nodular surface with “rock garden” findings (Figure 3C). A dynamic chest CT was performed showing tracheal stenosis during inspiration with an area of 1.5 cm^2^ (Figure 4A), which decreased to 0.8 cm^2^ (Figure 4C) during expiration compared with control (superior third of the trachea) with areas of 3.6 cm^2^ (Figure 4B) and 3.2 cm^2^ (Figure 4D), respectively. Bronchoscopy (Figure 5) found solid nodular lesions on the cartilaginous rings, with altered tracheal structure, especially in the superior and middle third throughout the main bronchi. A positron emission tomography scan was performed due to the initial finding of 8- to 10-mm nodules in the right lower lobe, where no metabolic activity was shown.

### Case 4

**Figure 6.**
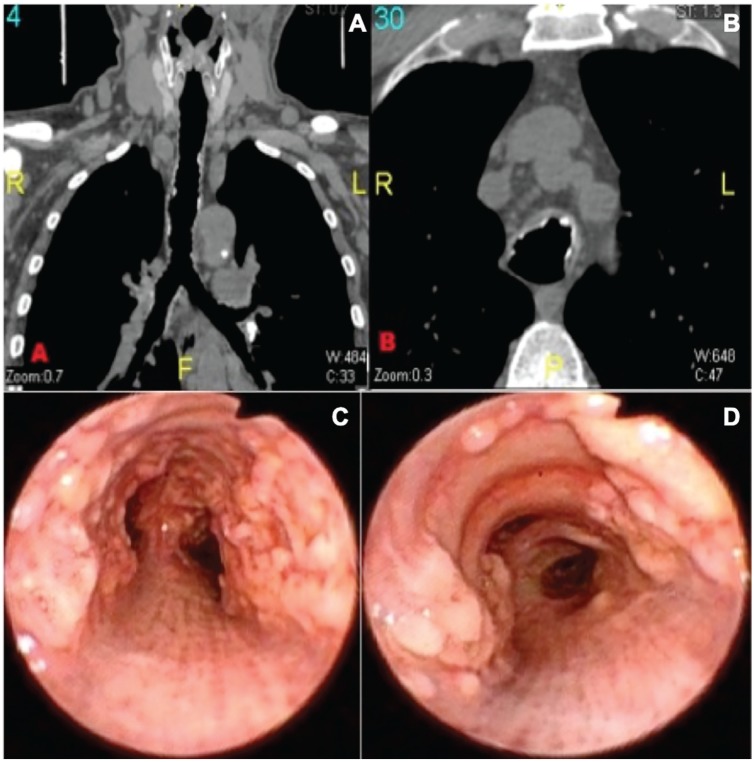
(A) Thoracic coronal computed tomography (CT) with a mild beaded appearance of the trachea with no airway stenosis. (B) Thoracic axial CT scan showing mild thickening of the anterolateral walls of the trachea with nodules, which are calcified. (C and D) Bronchoscopy showing diffuse submucosal nodules with an intact overlying mucosa, compromising the anterolateral walls and extending to the main bronchi. Observe the intact membranous trachea.

**Figure 7.**
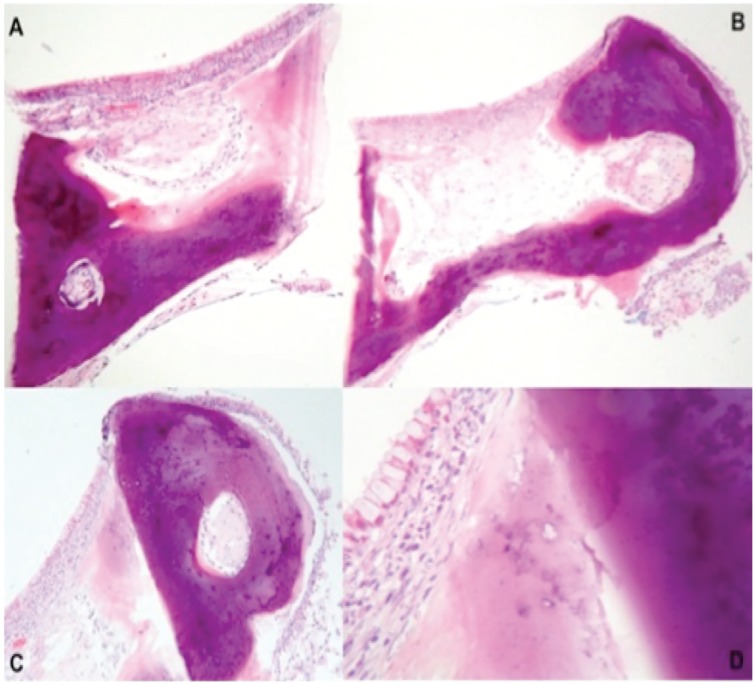
Biopsy taken during bronchoscopy. (A-C) Respiratory epithelium with submucosal osseous nodules and hematopoietic tissue formation. (D) Ciliated respiratory epithelium, global cell, and transition area between osseous and cartilaginous tissue.

A 61-year-old man, admitted due to an 8-month productive cough with hemoptysis, accompanied by low-grade fever, night sweating, diarrhea, and weight loss. He had a close family member who died from multidrug-resistant TB, and no prior personal history. Physical examination was unremarkable, except for a right supraclavicular nodule. Laboratory studies showed positive HIV and Mantoux tests. Chest CT was performed showing right paratracheal and bilateral pulmonary hiliar adenomegalies, along with thickening of the anterolateral tracheal walls (Figure 6A and B). Bronchoscopy showed diffuse submucosal nodules located on the proximal trachea, down to the main bronchi and the anterolateral walls, without affection of the membranous tracheal wall. Tracheal mucosa was normal (Figure 6C and D). Biopsies were performed and TO was confirmed (Figure 7).

### Case 5

**Figure 8.**
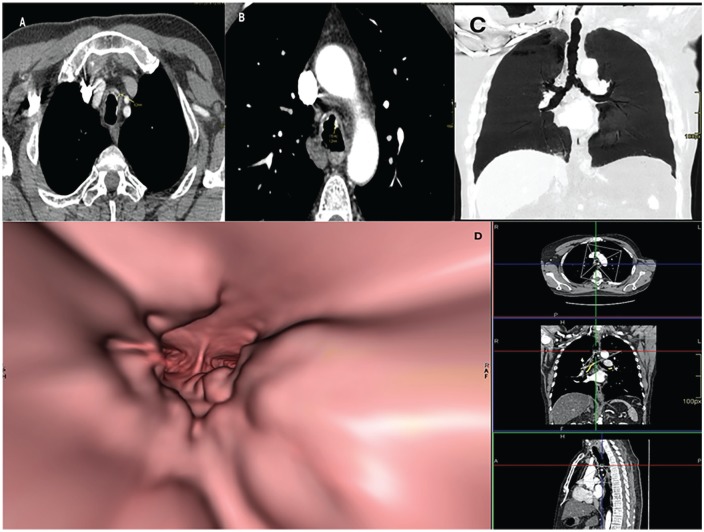
(A) Thoracic axial computed tomography (CT) scan with moderate nodular thickening of the anterior and lateral walls of the trachea of heterogeneous density and scalloped edges. Note the absence of compromise of the posterior wall of the trachea. (B) Thoracic axial CT scan with multiple calcified endotracheal nodules (170 HU). (C) Thoracic coronal CT scan maximal intensity projection reconstruction showing endobronchial nodules and beaded appearance of the trachea. (D) Virtual bronchoscopy showing multiple nodules giving a cobblestone appearance with compromise extending to the level of the tracheal carina, as shown by the axial, coronal, and sagittal reconstructions at the right.

A 56-year-old man with stage III melanoma, who had resection of the lesion plus lymph node drainage, without any other personal background nor smoking history. As part of the extension studies, a chest CT was performed in which metastasis was ruled out and TO was suspected. Chest axial CT scan shows thickening of the anterior and lateral tracheal walls of heterogeneous density and scalloped edges, along with calcified nodules (Figure 8A and B). On maximal intensity projection reconstruction, multiple endobronchial nodules with “beaded” appearance of the trachea were found (Figure 8C). In the virtual bronchoscopy, multiple nodules with “cobblestone throat” appearance were seen throughout the carina (Figure 8D).

A total of 33 patients diagnosed with TO between 2009 and 2019 were studied. Data were collected regarding clinical, imaging, and endoscopic findings.^2-27^

### Demographics

Twenty-five patients were males (76%) and 8 patients were females (24%). Age ranged between 20 and 79 years, with a mean age of 50 years. Most of the patients were between 60 and 69 (27%) years of age. Smoking status information was available for 23 patients, from which 20 (87%) patients were nonsmokers.

### Personal History

Out of 33 patients studied, medical history was reported for 28 patients, of whom only 1 patient had no personal history. Four patients had been diagnosed with asthma, 2 with chronic pulmonary obstructive disease and 14 had a history of pulmonary infection (Table 1). Of the latter, 3 patients had a history of recurrent pulmonary infections. Out of the 28 patients, 18 patients had a history of cardiovascular, oncologic, cirrhosis, chronic hepatitis B, gastroesophageal reflux disease, inguinal hernia, among other diseases.

**Table 1.** Personal History.

Characteristics	No. of Patients (%)
Asthma	4 (14)
COPD	2 (7)
Pulmonary infections^a^	20 (71)
Cardiovascular disease	5 (18)
Oncologic disease	4 (14)
Others (unrelated)	9 (32)
Healthy subject	1 (4)

Abbreviation: COPD, chronic pulmonary obstructive disease.

a Fifty-seven percent of patients had pneumonia, and 43% of patients received treatment for tuberculosis.

### Clinical Manifestations

Only 3 patients were asymptomatic. The most common manifestations were dyspnea on exertion (14 patients), chronic dry cough (14 patients), chronic productive cough (7 patients), hemoptysis (7 patients), and hoarseness (6 patients; Table 2).

**Table 2.** Clinical Manifestation at Diagnosis.

Clinical Manifestations	No. of Patients (%)
Dyspnea on exertion	14 (42)
Chronic dry cough	14 (42)
Chronic productive cough	7 (21)
Chest pain	2 (6)
Stridor/respiratory failure	2 (6)
Hoarseness	6 (18)
Hemoptysis	7 (21)
Fever	1 (3)
Asymptomatic	3 (9)
Others^a^	4 (12)

a Patients consulted for weight loss, fatigue, nausea, or abdominal pain.

### Physical Examination

Twenty-one patients (64%) had an unremarkable physical examination. Other findings were wheezing (5 patients, 15%), crackles (4 patients, 12%), stridor (3 patients, 9%), and respiratory failure (1 patient 3%).

### Localization of TO

Most of the patients had tracheal (29 patients) and main bronchi (17 patients) lesions (Table 3). Of the latter, only 1 patient had a single lesion on the left main bronchus. Two other patients had single lesions; one in the left vocal cord and another one in the proximal trachea, both causing severe stenosis.

**Table 3.** Tracheobronchopathia Osteochondroplastica Location.

Location	No. of Patients (%)
Larynx	8 (24)
Trachea	29 (88)
Main bronchi	17 (52)

### Findings on Chest CT Scan

Calcified submucosal nodules were reported in 30 cases. Nine patients had irregular tracheal walls with beaded appearance, 8 patients had tracheal stenosis, and 3 patients had deformed cartilage rings. Interestingly, 1 patient had a normal chest CT scan (Table 4).

**Table 4.** Thoracic Computed Tomography (CT) Findings.

Findings	No. of Patients (%)
Calcified submucosal nodules	30 (91)
Irregular tracheal walls with beaded appearance	9 (27)
Deformed and thickened cartilage rings	3 (9)
Tracheal stenosis	8 (24)
Single lesion	4 (12)
Image reported as normal	1 (3)

### Endoscopic Findings

Twenty-four patients had diffuse cartilaginous nodules protruding into the tracheal lumen giving a “cobblestone throat” appearance. Only 6 patients had a deformed and stenotic airway causing airflow obstruction (Table 5).

**Table 5.** Endoscopic Findings.

Findings	No. of Patients (%)
Whitish lesions throughout the tracheal mucosa, with mucosa hyperemia and edema (stage I)	0 (0)
Diffuse cartilaginous nodules protruding to the lumen giving a cobblestone appearance (stage II)	24 (80)
Deformed and stenotic airway that causes airflow obstruction (stage III)	6 (20)

## Discussion and Conclusions

Tracheobronchopathia osteochondroplastica is an idiopathic and probably underdiagnosed disease that causes tracheal stenosis. It is characterized by the presence of multiple submucosal cartilaginous and osseous nodules in the anterolateral walls of the tracheobronchial tree, cartilaginous rings, and sparing the posterior wall.^2-4^ Previous literature reviews had reported no gender preference; however, in this set of patients 76% were male. Similarly to prior studies, the most common age of diagnosis was between 60 and 80 years.

Clinical manifestations range from asymptomatic to severe airway obstruction, sometimes mimicking difficult to treat asthma. Thus, it is important for physicians to consider TO in the differential diagnosis in patients presenting chronic cough, hemoptysis, dyspnea on exertion, and recurrent pulmonary infections, which probably occur due to impaired mucociliary clearance.^2,3^ It has not yet been confirmed if this latter pathophysiologic mechanism represents a cause or a consequence of TO.^3,4^ There are multiple studies that report TO in patients with immune deficiencies such as immunoglobulin A deficiency, which could support the theory of an infectious etiology.^3^ Of the 33 patients described, 71% had a prior diagnosis of pulmonary infection: 57% had pneumonia and 43% had received or were on treatment for pulmonary TB. After diagnosis of TO, several patients were taken off anti-TB medications and antibiotics. Hence, an adequate TO diagnosis can avoid unnecessary interventions.

All TO diagnoses were made incidentally, which confirms the fact that physicians do not usually consider TO as a possible diagnosis. In this set of patients, only 9% were completely asymptomatic and 12% presented unrelated symptoms such as weight loss, malaise, or abdominal pain. The rest of the patients presented dyspnea on exertion, chronic dry cough, chronic productive cough, hemoptysis, and hoarseness. Importantly, 6% presented stridor and respiratory failure. As described above, the severity of clinical manifestations can range from asymptomatic to respiratory insufficiency and this will depend on the degree of tracheal stenosis. Furthermore, physical examination was unremarkable in 64% of the patients, similarly to that reported in the literature.^4,6^

There are multiple differential diagnoses for TO: tracheobronchial amyloidosis, atrophic polychondritis, and granulomatous diseases such as TB or sarcoidosis.^2^ In general, these diseases tend to involve the whole circumference of the tracheobronchial tree affecting the posterior wall and the overlying mucosa.2-4 Similarly, TO could also be confused with tracheobronchomalacia, in which the trachea and main bronchi lose their normal stiffness and the airway collapses during expiration. Therefore, a differential diagnosis can be made with dynamic CT.^7^

Due to the development of imaging technologies, diagnosis has improved. Although chest radiograph is usually unremarkable in these patients, changes such as tracheal scalloping and nodular irregularity have been reported.^28^ Thoracic CT and bronchoscopy tend to show pathognomonic signs of the disease.

Regarding chest CT, a “beaded” appearance of the airway might be seen^29^ (Figure 8C) due to the presence of cartilaginous submucosal nodules, with or without calcifications (Figure 8B and Figure 2, respectively), that protrude to the tracheal lumen in the anterolateral walls.^30^ The lower third of the trachea is commonly affected, although it can extend through the main bronchi, where cartilaginous rings are still found.^11^ Consistently, 88% of the patients had tracheal diseases, while 52% had lesions on the main bronchi and 24% on the larynx. The membranous posterior wall is always spared, and cartilage rings appear thickened and deformed without evidence of external compression^28,30^ (Figures 6B and 8A). In this set of patients, 4 patients presented a unique form of the disease, consisting of a single lesion described as a mass that obstructed the airway: 2 patients on the larynx, 1 patient on the trachea, and 1 patient on the main bronchi leading to atelectasis. The limitation of CT scans lies in its suboptimal sensitivity, especially in the earlier stages of the disease where nodules are <2 mm and calcification is less common.^26,30^

Although dynamic CT and virtual bronchoscopy are not routinely performed, in our series, one dynamic CT showed a severe stenosis in the middle section of the trachea as described above (Figure 4). Regarding virtual bronchoscopy, it is an imaging technique that allows a noninvasive visualization of the superior airway. However, this does not allow for the analysis of the mucosa or biopsy; thus, it does not replace classic bronchoscopy.^27^ We report 2 virtual bronchoscopies in which we were allowed to visualize endotracheal nodules with the pathognomonic “cobblestone” appearance (Figures 3C and 8D).

Bronchoscopy is considered the gold standard for diagnosis; multiple whitish and hard osseocartilaginous nodular lesions are seen in the anterolateral walls of the trachea, giving a “rock garden” or “paving stone” appearance.^8,26,30^ Nodule size oscillates between 1 and 10 mm; in most cases, these are found in the distal two thirds of the trachea.^29,30^ A study made from a large Chinese cohort described 3 stages of the disease based on the severity of presentation on bronchoscopy findings: stage I (plaque-like infiltrations of soft whitish lesions throughout the mucosa, with mucosal hyperemia, and edema), stage II (diffuse cartilaginous nodules protruding to the lumen giving a “cobblestone” appearance), and stage III (deformed and stenotic airway that may cause airflow obstruction; Figure 5).^31^ These bronchoscopy stages can be easily correlated with the imaging findings on chest CT; one of our patient was classified as stage I because lesions found were around 1 mm and scattered throughout the airway (Figure 3). Similarly to findings on chest CT: 1 soft tissue nodule (Figure 2). Moderate disease is classified as stage II and it was the most common presentation in the literature review (80%) as well as in our cases (Figures 1, 6C and D, and 8D). The pathognomonic “cobblestone” or “rock garden” appearance is due to thickening of the anterolateral tracheal walls. Nodules are bigger and may have some degree of calcification, which can be seen on chest CT (Figures 6B, and 8A and B). Last, 20% of the patients reviewed from the literature and 1 patient in our series presented severe stenosis (stage III on bronchoscopy; Figure 5), which was confirmed by dynamic CT that found a 75% stenosis in the middle section of the trachea (Figure 5).

On the other hand, the need for histopathology is still controverted,^30^ as visual detection of the nodules is usually sufficient for diagnosis. In any case, some authors still consider biopsies to be necessary in order to confirm the diagnosis and assess for associated conditions.^26,30^ Histopathologic findings were epithelial squamous metaplasia along with submucosal nodules in cartilaginous areas; some with ossification, calcification, or hematopoietic bone marrow in them,^10^ as can be seen in Figure 6.

Treatment for TO is unspecific and symptomatic. In many cases, a combination of antitussives, inhaled bronchodilators, steroids, antibiotics, or clearance therapies are used^1^ to manage mucociliary impairment secondary to squamous metaplasia and loss of normal airway structure.^26^ Even though the progression of the disease is benign due to its long-term stability (with studies showing minimal progression after 20 years of diagnosis), a minority of patients can present severe airway obstruction that would require local treatment such as laser, dilation, resection of the nodules, or even tracheal resection.^4,5,26^

In conclusion, TO is a rare and unknown disease that should be considered in patients with chronic cough, dyspnea, and recurrent pulmonary infections.^2,3^ We believe chest CT and bronchoscopy are both ideal diagnostic approaches, the latter being the gold standard. Therefore, it is crucial for physicians, especially radiologists and endoscopists, to be well acquainted regarding TO. Histopathology studies are rarely necessary since imaging and endoscopic findings are pathognomonic. Accurate diagnosis can avoid unnecessary procedures in patients who had been previously misdiagnosed.
